# Preemptive ganciclovir for mechanically ventilated patients with cytomegalovirus reactivation

**DOI:** 10.1186/s13613-020-00793-2

**Published:** 2021-02-11

**Authors:** Laurent Papazian, Samir Jaber, Sami Hraiech, Karine Baumstarck, Sophie Cayot-Constantin, Nadia Aissaoui, Boris Jung, Marc Leone, Bertrand Souweine, Carole Schwebel, Jérémy Bourenne, Jérôme Allardet-Servent, Toufik Kamel, Qin Lu, Christine Zandotti, Anderson Loundou, Christine Penot-Ragon, Jean Chastre, Jean-Marie Forel, Charles-Edouard Luyt, the Preemptive Herpesviridae Treatment Study Group, REVA Network

**Affiliations:** 1 Médecine Intensive Réanimation, Aix-Marseille Université, Hôpital Nord, Chemin des Bourrely, 13015 Marseille, France; 2 Réanimation Chirurgicale, Centre Hospitalier Universitaire de Montpellier, Hôpital St-Eloi, Montpellier, France; 3 Laboratoire de Santé Publique, Aix-Marseille Université, Assistance Publique–Hôpitaux de Marseille, Marseille, France; 4 Département de Médecine Periopératoire, CHU Clermont-Ferrand, Clermont-Ferrand, France; 5 Médecine Intensive Réanimation, Hôpital Européen Georges-Pompidou, APHP, Paris, France; 6 Médecine Intensive Réanimation, Centre Hospitalier Universitaire de Montpellier, Hôpital Lapeyronie, Montpellier, France; 7 Service d’Anesthésie-Réanimation, Aix-Marseille Université, Hôpital Nord, Assistance Publique–Hôpitaux de Marseille, Marseille, France; 8 Réanimation Médicale, CHU Gabriel-Montpied, Clermont-Ferrand, France; 9 Médecine Intensive Réanimation, CHU Grenoble Alpes, La Tronche, France; 10 Réanimation des Urgences et Médicale, Aix-Marseille Université, Hôpital Timone, APHM, Marseille, France; 11 Réanimation, Hôpital Européen, Marseille, France; 12 Médecine Intensive Réanimation, Centre Hospitalier Régional, Orléans, France; 13 Réanimation Chirurgicale Polyvalente, Département d’Anesthésie-Réanimation, Hôpitaux Universitaires Pitié Salpêtrière-Charles Foix, APHP, Paris, France; 14 Laboratoire de Virologie, IHU Méditerranée Infection, CHU Timone UMR190-Emergence des Pathologies Virales, Marseille, France; 15 Pharmacie, Hôpitaux Sud, APHM, Marseille, France; 16 Sorbonne Université, INSERM, Médecine Intensive Réanimation, Institut de Cardiologie, Hôpitaux Universitaires Pitié Salpêtrière-Charles Foix, Assistance Publique–Hôpitaux de Paris, Paris, France

**Keywords:** Mechanical ventilation, Randomized, Clinical trial, Mortality, Immunocompetent

## Abstract

**Background:**

The effect of cytomegalovirus (CMV) reactivation on the length of mechanical ventilation and mortality in immunocompetent ICU patients requiring invasive mechanical ventilation remains controversial. The main objective of this study was to determine whether preemptive intravenous ganciclovir increases the number of ventilator-free days in patients with CMV blood reactivation.

**Methods:**

This double-blind, placebo-controlled, randomized clinical trial involved 19 ICUs in France. Seventy-six adults ≥ 18 years old who had been mechanically ventilated for at least 96 h, expected to remain on mechanical ventilation for ≥ 48 h, and exhibited reactivation of CMV in blood were enrolled between February 5th, 2014, and January 23rd, 2019. Participants were randomized to receive ganciclovir 5 mg/kg bid for 14 days (*n* = 39) or a matching placebo (*n* = 37).

**Results:**

The primary endpoint was ventilator-free days from randomization to day 60. Prespecified secondary outcomes included day 60 mortality. The trial was stopped for futility based on the results of an interim analysis by the DSMB. The subdistribution hazard ratio for being alive and weaned from mechanical ventilation at day 60 for patients receiving ganciclovir (*N* = 39) compared with control patients (*N* = 37) was 1.14 (95% CI from 0.63 to 2.06; *P* = 0.66). The median [IQR] numbers of ventilator-free days for ganciclovir-treated patients and controls were 10 [0–51] and 0 [0–43] days, respectively (*P* = 0.46). Mortality at day 60 was 41% in patients in the ganciclovir group and 43% in the placebo group (*P* = .845). Creatinine levels and blood cells counts did not differ significantly between the two groups.

**Conclusions:**

In patients mechanically ventilated for ≥ 96 h with CMV reactivation in blood, preemptive ganciclovir did not improve the outcome.

## Introduction

It is generally reported that 60 to 80% of immunocompetent adults are human cytomegalovirus (CMV) seropositive [1, 2]. Following primary infection (asymptomatic or with nonspecific signs and symptoms), CMV remains quiescent in monocytes and macrophages in multiple organs (latency). Due to immune status alterations, critically ill patients are at risk of reactivation [3]. A recent meta-analysis of 18 studies involving 2398 immunocompetent patients showed that 31% of intensive care unit (ICU) patients may experience CMV reactivation, regardless of the site (blood or lung) of reactivation [4]. CMV reactivation may cause injury by different mechanisms: by a direct cytopathologic effect in organs containing the virus, such as occurs during CMV pneumonia [5–7]; by an excessive immune response to the virus, such as what is hypothesized to happen in ARDS patients [8]; and/or by altering the immune response facilitating fungal and bacterial infections [9–12]. Regarding the pro-inflammatory properties of CMV, it has been shown that CMV can enhance the progression of postaggressive lung fibrosis [13]. However, there is no convincing data to support the use of antiviral treatment when CMV is detected in non-immunocompromised critically ill patients. The pathogenicity of CMV in immunocompetent critically ill patients is also questionable because based on epidemiological noninterventional studies, reactivation might only be an indicator of immune response impairment and illness severity not necessitating diagnostic procedures and treatment. An interventional trial aiming to determine whether antiviral therapy is safe and effective for preventing CMV reactivation showed that valacyclovir or low-dose valganciclovir were able to decrease CMV reactivation in a general population of critically ill patients [14]. The duration of mechanical ventilation was not assessed, and increased mortality was reported for the valacyclovir group [14]. A multicenter double-blind, placebo-controlled, randomized clinical trial conducted in 160 CMV-seropositive adults with either sepsis or trauma and respiratory failure concluded that the prophylactic use of ganciclovir was able to reduce CMV reactivation and increase the number of ventilator-free days, but was unable to lower interleukin-6 levels (main objective) [15]. To reduce the number of patients receiving ganciclovir, which is a drug associated with some side effects, it would be of interest to limit the administration of this treatment to patients presenting reactivation without any sign of end-organ disease. The present study was therefore designed to assess whether preemptive ganciclovir is able to increase the number of ventilator-free days in patients with CMV reactivation in blood after at least 4 days of invasive mechanical ventilation.

## Materials and methods

### Study design

This randomized, multicenter, double-blind, placebo-controlled trial was conducted in 19 ICUs in France (from February 5th, 2014, to January 23rd, 2019) (Trial registration—ClinicalTrials.gov Identifier NCT02152358). The sponsor was the Assistance Publique–Hôpitaux de Marseille, and a grant was obtained from the French Ministry of Health (PHRC 2011). An independent Ethics Committee (Comité de Protection des Personnes Sud Méditerranée 5) approved the protocol and the amendments. Study sites and investigators are listed in the Appendix. The trial protocol has already been published (Additional file 1) [16].

During the study period, potentially eligible patients (i.e., those under invasive mechanical ventilation for ≥ 96 h) were screened twice weekly for CMV and herpesvirus simplex (HSV) reactivations, respectively, with quantitative or qualitative polymerase chain reactions (PCR) on whole blood or oropharyngeal swabs collected the same day for as long as the patients remained under invasive mechanical ventilation with a maximum of 30 days of mechanical ventilation. Patients with CMV blood reactivation or concomitant HSV oropharyngeal reactivation and CMV blood reactivation were eligible for the CMV trial, whereas patients with HSV oropharyngeal reactivation were eligible for the HSV trial. The results of the study related to HSV-positive patients (HSV trial) have been recently published [16]. We report here the results of the CMV trial. Once the patient was included in the trial, the systematic PCR results were no longer transmitted to the clinicians (unless the clinician specifically asked for them when there was a suspected active infection). Patients could not be included in another study. Patients and/or their relatives were informed of this screening until December 2015, when screening became routine care, and French law rendered informing them no longer necessary.

### Study participants

Patients who were at least 18 years of age, had been mechanically ventilated for at least 96 h with a predicted mechanical ventilation duration longer than 48 h, had CMV-positive whole blood and provided written informed consent from the patient or his/her legally authorized representative were eligible for enrollment. Finally, the patient’s follow-up informed consent was obtained as soon as possible. Exclusion criteria were as follows: age < 18 years; patients deprived of freedom or under legal protection; patients not covered by social security; use of acyclovir, ganciclovir or another antiviral with anti-HSV/CMV activity (e.g., cidofovir or foscarnet) at the time of randomization; patients with known hypersensitivity to ganciclovir; patients who had an active HSV or CMV infection treated during the preceding month; patients who were pregnant or lactating; patients with pancytopenia, neutropenia ≤ 500/mm^3^, or thrombocytopenia < 25 G/L; patients with solid-organ or bone marrow transplants; patients on immunosuppressant therapy (including corticosteroids at ≥ 0.5 mg/kg/day of prednisone or its equivalent for > 1 month); patients with human immunodeficiency virus infection; patients with moribund conditions defined as a preinclusion Simplified Acute Physiology Score (SAPS) II ≥ 75; patients regarding whom a decision had been made to withhold or withdraw life-sustaining treatment; and patients with an ICU readmission during the same hospital stay.

### DNAemia

“DNAemia” was defined as the detection of CMV DNA in whole blood. The nucleic acid amplification techniques used in all sites were calibrated to a standard calibrator, which was the WHO International Standard for Human CMV [17]. A threshold set at 500 IU/mL whole blood was chosen as the inclusion criterion (close to the limit of detection at the time the study was designed).

### Randomization

A centralized, secure, web-based, randomization system using minimization assigned patients at a 1:1 ratio, with stratification by study site, prerandomization invasive mechanical ventilation duration (≤ 14 or > 14 days) and number of organ failure(s) split into 2 levels: < 2 or ≥ 2 organ failures according to the SeqSequential Organ-Failure Assessment [18] (SOFA) score. A specific organ failure was defined by the corresponding SOFA score > 2.

### Study interventions

Patients were randomized to receive ganciclovir administered intravenously in 1 h at a dose of 5 mg/kg or a matching placebo (control group) preparation every 12 h for patients with normal renal function (creatinine < 120 µmol/l) and for a maximum total duration of 14 days. For extubated patients discharged from the ICU before day 14 post-randomization, the study agent was stopped at discharge. Ganciclovir doses were adjusted to renal function according to the manufacturer’s recommendations. Placebo and ganciclovir were conditioned in similar bottles that were distributed post-randomization and reconstituted in glucose solution by the nurses before each administration. By February 2016, 37 patients had been included, and the placebo-batch dates had surpassed the expiration. The independent Ethics Committee approved trial modification of the placebo- and ganciclovir-distribution procedure. To maintain the blinded-study design for ICU personnel, the hospital pharmacy or a nurse from a different unit reconstituted and distributed ganciclovir- or placebo-containing glucose bags daily to ICU nurses treating the patients throughout the study period. Criteria to interrupt the ganciclovir treatment or its placebo were the presence of leukopenia < 1000/mm^3^ and/or neutropenia ≤ 500/mm^3^ and/or thrombocytopenia < 25 G/L.

### Study outcomes

The primary outcome was the number of ventilator-free days at day 60 (VFD60) from randomization, i.e., days alive and free from invasive mechanical ventilation [19]. For patients who died before day 60, that number was zero, regardless of invasive mechanical ventilation duration. For patients with multiple mechanical ventilation episodes during the 60-day follow-up period, days without invasive mechanical ventilation were considered only after the last weaning-off invasive mechanical ventilation. Secondary outcomes included the day-60 mortality rate; mechanical ventilation duration; occurrence of HSV bronchopneumonitis or active cytomegalovirus infection; secondary bacterial pneumonia, bacteremia or fungemia; incidence of acute respiratory distress syndrome (ARDS) and septic shock post-randomization. Active CMV infection was defined by the presence of an antigenemia > 10 cells/200 000 cells and/or a positive qualitative PCR on BAL and/or the presence of IgM WITH associated clinical or biological symptoms (transaminases > 3 times the norm) (digestive = positive biopsy). The main safety endpoints were myelotoxicity and acute renal failure.

### Data safety monitoring board

Safety oversight was under the direction of an independent Data Safety Monitoring Board (DSMB). The DSMB convened by teleconferencing or in person at 25%, 50% and 75% of enrollment to review adverse events or earlier if so needed. The DSMB might be questioned for other logistical, ethical, and clinical points.

### Statistical analysis

According to a previous study evaluating *Herpesviridae* reactivation in patients with prolonged invasive mechanical ventilation, the expected standard deviation (SD) of ventilator-free days for controls was ± 20 days [20]. We hypothesized that preemptive ganciclovir administration could increase the number of ventilator-free days by 8 days. To have 80% power with a 5% alpha level, 112 patients had to be included per group (after applying a correcting coefficient of 0.864 to adjust for asymptotic test efficiency). To account for potential losses to follow-up, that number was raised to 120 per group, meaning 240 patients had to be included. The statistical analysis plan specific to the CMV arm detailed all the scheduled methodological approach (Additional file 2).

The primary outcome was first analyzed according to the Fine & Gray competitive risk model [21] (primary analysis), with death as the competitive risk. The results are presented as the subdistribution hazard ratio (SHR) and the 95% confidence interval (95% CI) effect size. Complementary results (secondary analyses) were provided for the primary outcome: (i) VFD60 median comparison between groups (Mann–Whitney test); (ii) hierarchical composite endpoint (alive and ventilator free) that considers death worse than prolonged ventilation and compares each patient with every other patient in a win–lose–tie for each comparison (Mann–Whitney test) [22–24].

Data are expressed as the median [interquartile range (IQR)] or mean (± standard deviation, SD), as appropriate. Between-group comparisons used Student’s t-test or the Mann–Whitney U-tests for continuous variables and χ^2^ or Fisher’s exact tests for categorical variables. Censored outcomes (time to death and time to weaning-off mechanical ventilation) were described with the Kaplan–Meier method, with between-group log-rank–test comparisons. The main analyses were conducted on an intention-to-treat basis. All analyses were computed with IBM SPSS statistics 20, R software, version 3.5.1 (R Foundation) at a two-sided, 5% alpha level.

Non-prespecified metanalyses were finally performed for three endpoints taking into account two already published randomized clinical trials [14, 15] and the present one. Day 28 mortality and hospital mortality were analyzed using odd ratios (95% CI), defined as the ratio of the probability of an event occurring between two groups. Ventilator-free days to day 28 were analyzed using standardized means difference (standard error), expressing the size of the intervention effect (means and standard deviations were estimated from the medians and interquartile ranges) [25, 26]. We used fixed effects [27] and random effects models [28], which account for the between‐study heterogeneity by weighting studies similarly. Heterogeneity was assessed using the I^2^ statistic, which represents the percentage of variance due to between‐study factors rather than to sampling error [29]. We considered values of I^2^ > 50% as indicative of large heterogeneity.

## Results

**Fig. 1:**
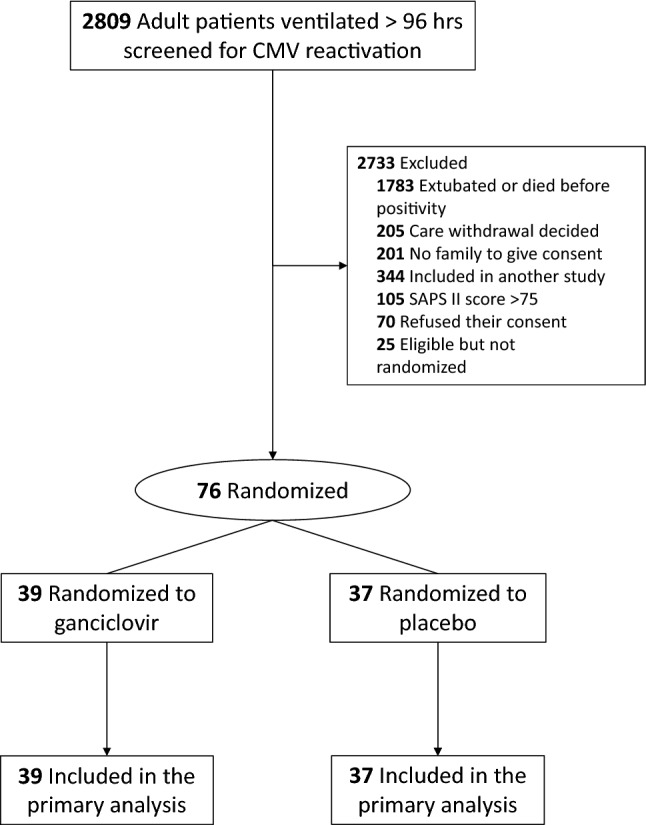
Flowchart of the study

Due to a large gap between the theoretical and real inclusion curves and also the budget implications, the sponsor solicited the independent DSMB. The DSMB, in agreement with the sponsor, asked therefore to have results for the primary endpoint. Based on this report, the DSMB wanted unblinding to determine continuation or to stop the study. After breaking the randomization code, the DSMB recommended to stop the trial for futility after the interim analysis had concluded that at least 822 patients would have been necessary to show a difference between the two groups. The trial was therefore stopped on May 15th, 2019. Among the 2809 patients screened for HSV and cytomegalovirus, 76 were randomized (Fig. 1). There was no consent withdrawal, and all 76 patients remained in the analysis: 39 ganciclovir recipients and 37 placebo-treated controls. The baseline characteristics at ICU admission (Table 1) did not differ between the two groups. Their characteristics at randomization were also comparable (Table 2). The median [IQR] prerandomization duration of mechanical ventilation was 15.0 [10.0–22.0] days in the placebo group and 14.0 [9.0–22.0] days for the ganciclovir group (*P* = 0.925). The modified clinical pulmonary infection score at randomization was 5.8 ± 2.3 in the placebo group and 5.4 ± 2.4 in the ganciclovir group (*P* = 0.501). The median [IQR] quantity of CMV was 1760 [1031–3344] IU/ml in the placebo group compared with 1915 [1120–3105] IU/ml in the ganciclovir group (*P* = 0.763).

**Table 1:** Patient characteristics at ICU admission

Characteristics	Placebo group (*N* = 37)	Ganciclovir group (*N* = 39)
Age, *y*	67.0 (59.0–72.0)	63.0 (54.0–71.0)
Male sex, no. (%)	25 (67.6)	31 (79.5)
Body mass index, kg/m^2^^a^	27.7 (21.3–31.2)	26.2 (23.6–35.2)
McCabe score = 1 (non-fatal), no. (%)	32 (86.5)	28 (71.8)
Preexisting disease, no. (%)		
NYHA III/IV	4 (10.8)	3 (7.7)
Cancer/hemopathy	3 (8.1)	7 (17.9)
Diabetes mellitus	6 (16.2)	10 (25.6)
COPD	4 (10.8)	7 (17.9)
Cirrhosis	3 (8.1)	0 (0)
Chronic renal failure ^a^	1 (2.7)	2 (5.1)
Alcoholism, no. (%)	6 (16.2)	5 (12.8)
Recent transfusion (< 1 month), no. (%)	12 (32.4)	13 (33.3)
Corticosteroids (< 1 month), no. (%)	5 (13.5)	1 (2.6)
No preexisting disease and no risk factor, no. (%)	17 (45.9)	16 (41)
Admission category, no. (%)		
Medical	31 (83.8)	34 (87.2)
Emergency surgery	2 (5.4)	4 (10.3)
Planned surgery	4 (10.8)	1 (2.8)
Primary reason for mechanical ventilation, no. (%)		
Acute respiratory failure	13 (35.1)	12 (30.8)
Septic shock	8 (21.6)	8 (20.5)
Cardiogenic shock	2 (5.4)	5 (12.8)
Post-operative acute respiratory failure	4 (10.8)	1 (2.6)
Exacerbation of chronic respiratory disease	0 (0)	4 (10.3)
Trauma	1 (2.7)	0 (0)
Neurologic	1 (2.7)	1 (2.6)
Cardiac arrest	1 (2.7)	0 (0)
Others	7 (18.9)	8 (20.5)
SAPS II	45.0 (38.5–56.5)	45.0 (37.0–59.0)
SOFA score	10 (8–15)	9 (7–10)
Organ/system failure, no. (%)^b^		
Cardiovascular	23 (62.2)	28 (71.8)
Respiratory	26 (81.2)	29 (80.6)
Renal	11 (29.7)	11 (28.2)
Central nervous	8 (21.6)	1 (2.6)
Hepatic	1 (2.8)	2 (5.3)
Coagulation	4 (10.8)	2 (5.1)

Results are expressed as median (IQR) unless stated otherwise

NYHA denotes New York Heart Association, COPD chronic obstructive pulmonary disease, SAPS Simplified Acute Physiology Score, SOFA Sequential Organ-Failure Assessment

^a^ Creatinine clearance < 60 ml/min, creatinine > 150 µmol/liter or chronic dialysis

^b^ Organ/system failure was deemed present when the corresponding SOFA score was > 2. When data regarding organ/system failure were missing, number of assessable patients is reported

**Table 2:** Patient characteristics at randomization

Characteristics	Placebo group (*N* = 37)	Ganciclovir group (*N* = 39)
Ongoing antimicrobial treatment, no. (%)	26 (70.3)	28 (71.8)
ECMO use, no. (%)	5 (13.5)	6 (15.4)
Renal replacement therapy, no. (%)	14 (37.8)	13 (33.3)
SOFA score	8.0 (5.0–11.0)	8.5 (4.0–10.3)
Organ/system failure, no. (%)^a^		
Cardiovascular	18 (48.6)	19 (48.7)
Respiratory	22 (59.5)	24 (61.5)
Renal	12 (32.4)	14 (35.9)
Central nervous	6 (16.2)	4 (10.3)
Hepatic	2 (5.4)	3 (7.9)
Coagulation	2 (5.4)	2 (5.1)
Body temperature, ℃	37.7 (36.3–38.2)	37.8 (36.9–38.3)
White blood-cell count, G/L	13.6 (10.2–17.8)	14.0 (10.2–20.0)
Neutrophil count, G/L	11.4 (7.5–15.6)	10.7 (7.1–16.0)
PaO_2_/FiO_2_, mmHg	175.0 (127.5–237.0)	170.0 (111.0–240.0)
Radiologic score	6.0 (4.0–8.5)	5.5 (3.0–8.0)
Tidal volume, mL	450.0 (390.3–554.3)	448.0 (370.5–497.0)
Respiratory rate, cycles/min	28.0 (20.0–33.0)	25.0 (22.0–29.3)
Minute ventilation, L/min	12.0 (9.2–14.9)	10.4 (8.7–13.9)
PEEP, cmH2O	8.0 (5.0–10.0)	8.0 (6.0–12.0)
Plateau pressure, cmH2O	19.0 (15.0–26.0)	22.0 (17.3–26.5)
FiO_2_, %	40.0 (30.0–50.0)	40.0 (35.0–55.0)
pH	7.43 (7.36–7.48)	7,42 (7.37–7.48)
PaO_2_, mmHg	85.0 (70.5–102.0)	81.0 (67.5–115.0)
PaCO_2_, mmHg	35.0 (33.0–41.0)	40.5 (35.0–45.3)

Results are expressed as median (IQR) unless stated otherwise

There were no significant between-group differences in characteristics at randomization

MV denotes mechanical ventilation, ECMO extracorporeal membrane oxygenation, SOFA Sequential Organ-Failure Assessment, PaO_2_/FiO_2_ partial oxygen pressure in arterial blood/fraction of inspired oxygen ratio

^a^Organ/system failure was deemed present when the corresponding SOFA score was > 2

### Study drug

All patients received at least one study-agent dose. The median [IQR] treatment durations were similar (Table 3). Thirty-nine patients (18 ganciclovir recipients and 21 controls) stopped the study agent earlier than scheduled. Reasons for discontinuation for ganciclovir recipients and controls were 16 deaths (9 and 7), 15 ICU discharges (8 and 7), one ganciclovir/placebo-related adverse event (0 and 1), five cases of CMV reactivation/HSV bronchopneumonitis (4 and 1), and two physicians’ decisions (0 and 2).

**Table 3:** Outcomes

Parameters	Placebo group (*N* = 37)	Ganciclovir group (*N* = 39)	*P* Value
Primary outcome			
Ventilator-free days on day 60	0 (0–43)	10 (0–51)	0.459
Secondary outcomes (post-randomization)			
Day-60 mortality, no. (%)	16 (43.2)	16 (41.0)	0.845
Duration of MV	20 (7–40)	12 (6–29)	0.246
ICU length of stay (from admission)	44.0 (21.0–66.5)	36.0 (24.0–51.0)	0.377
ICU length of stay (from randomization)	26.0 (11.0–50.0)	17.0 (8.0–34.0)	0.318
Hospitalization length (from admission)	60.0 (33.0–75.5)	65.0 (28.0–78.0)	0.988
Hospitalization length (from randomization)	42.0 (18.5–60.0)	38.0 (13.0–60.0)	0.945
HSV bronchopneumonitis, no. (%)	1 (2.7)	0 (0)	0.487
Cytomegalovirus infection, no. (%)	5 (13.5)	1 (2.6)	0.103
Ventilator-associated pneumonia, no. (%)	15 (40.5)	13 (33.3)	0.515
Secondary bacteremia or fungemia, no. (%)	8 (21.6)	7 (17.9)	0.688
ARDS post-randomization, no. (%)	6 (16.2)	6 (15.4)	0.921
Mild^a^	0	0	
Moderate^a^	3	3	
Severe^a^	3	3	
Septic shock post-randomization, no. (%)	14 (37.8)	13 (33.3)	0.682
Renal replacement therapy until day 28, no. (%)	18 (48.6)	16 (41.0)	0.504
Number of days with study drug, no. (%)	14 (7.5–14)	14 (6.0–14)	0.991

Results are expressed as median (IQR) unless stated otherwise

MV denotes mechanical ventilation, HSV herpes simplex virus, ARDS acute respiratory distress syndrome, PaO_2_/FiO_2_ partial oxygen pressure in arterial blood/fraction of inspired oxygen ratio, PEEP positive end-expiratory pressure, CPAP continuous positive airway pressure

^a^The Berlin definition of ARDS is as follows: mild: PaO_2_/FiO_2_ > 200 but ≤ 300, with PEEP or CPAP ≥ 5 cm H_2_O; moderate: PaO_2_/FiO_2_ > 100 but ≤ 200, with PEEP or ≥ 5 cm H_2_O; severe: PaO_2_/FiO_2_ ≤ 100 with PEEP ≥ 5 cm H_2_O^20^

### Primary endpoint

The subdistribution hazard ratio for being alive and weaned from mechanical ventilation at day 60 after randomization in patients receiving ganciclovir compared with control patients was 1.14 (95% CI from 0.63 to 2.06; *P* = 0.66) in the primary analysis. The median [IQR] numbers of VFD60 after randomization for ganciclovir-treated patients and controls were 10 [0–51] and 0 [0–43] days, respectively (*P* = 0.46). Hierarchical composite endpoint (alive and ventilation free) using the alternative technique to compare VFD60 (Finkelstein method [23]) did not result in a significant between-group difference (*P* = 0.524). The post hoc power of the final analysis of the primary endpoint was 14.2%.

### Secondary endpoints

**Fig. 2:**
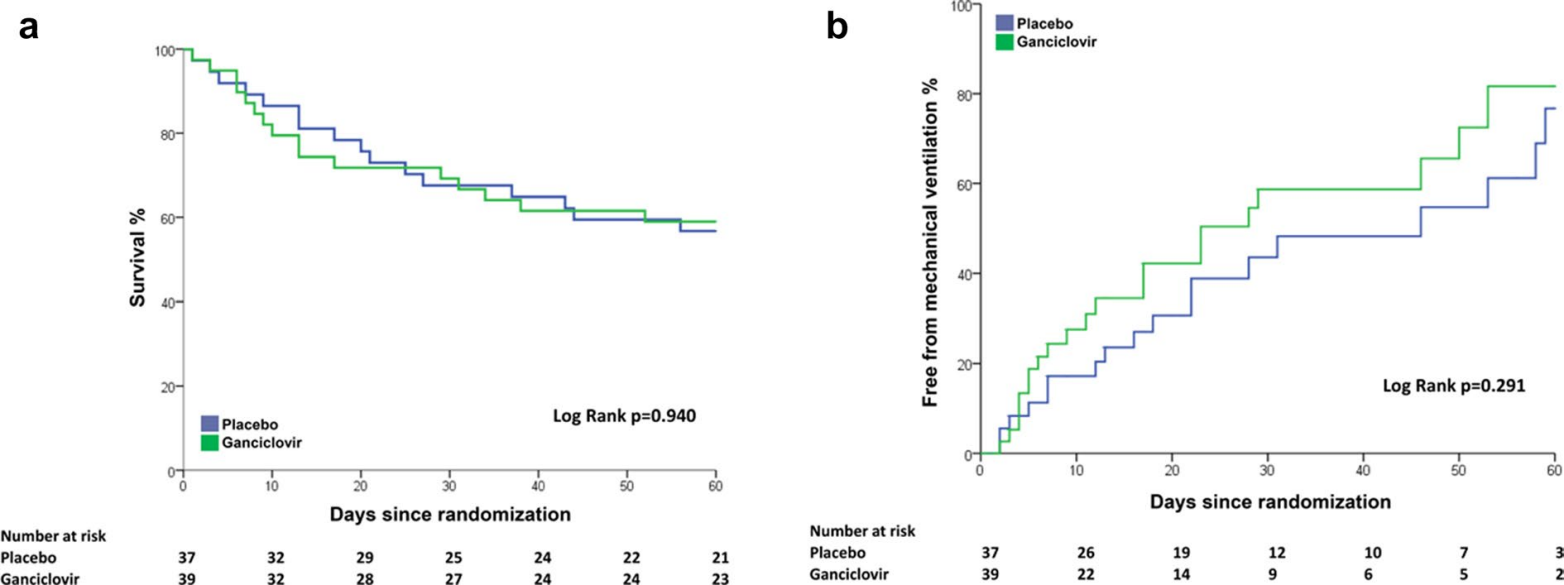
**a** Survival curves. **b** Cumulative proportion of patients weaned from invasive mechanical ventilation

**Fig. 3:**
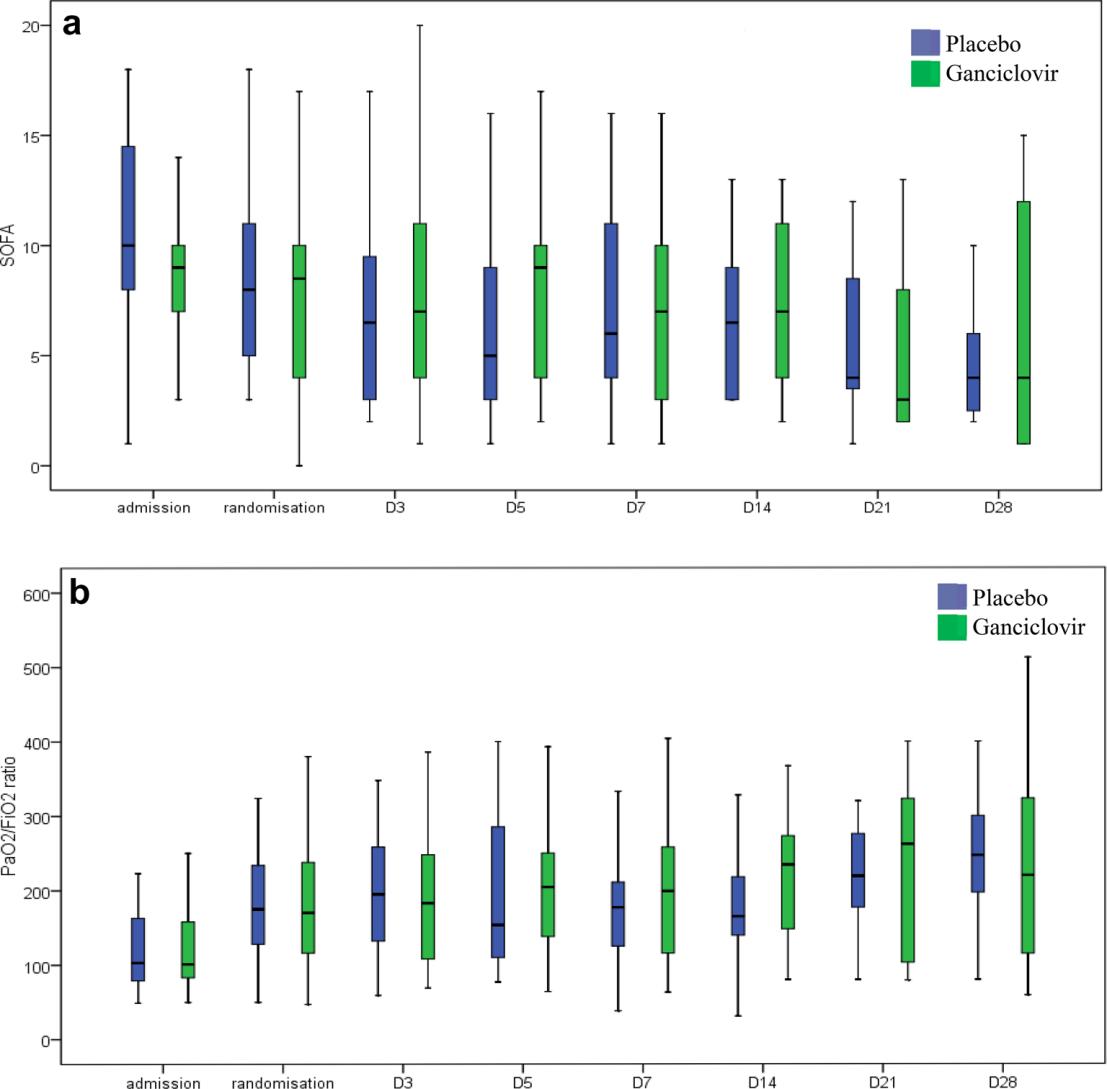
**a** Kinetics of the Sequential Organ-Failure Assessment (SOFA) score from Randomization to Day 28 According to Study Group. **b** Evolution of PaO2/FiO2 ratio from Randomization to Day 28 According to Study Group. PaO2/FiO2 denotes partial oxygen pressure in arterial blood/fraction of inspired oxygen ratio

As shown in Table 3 and in Fig. 2a, there was no difference between the two groups regarding mortality evaluated at day 60 after randomization. All patients died in the ICU after a median [IQR] length of stay after randomization of 11.5 [6.3–30.5] days in the ganciclovir group and 18.5 [7.5–34.5] days in the control group (*P* = 0.46). On day 60, 18 (46%) ganciclovir recipients and 19 (51%) controls had died or were still on mechanical ventilation (*P* = 0.65). The median [IQR] duration of mechanical ventilation after randomization was 12 [6–29] days for all patients from the ganciclovir group and 20 [7–40] days for controls (*P* = 0.25) (Fig. 2b). The median [IQR] duration of mechanical ventilation for day 60 survivors was 12 [5–29] days after randomization for ganciclovir recipients and 22 [7–55.5] days for controls (*P* = 0.31). Other secondary endpoints (Table 3) did not differ between groups. There was no difference between the two groups regarding either the incidence or the microorganisms causing bacteremia/fungemia and ventilator-associated pneumonia diagnosed after inclusion (Table 3 and in the Additional file 3: Tables S1 & S2). An active CMV infection was diagnosed in 5 patients from the placebo group (after a median of 14 days from inclusion and a range from 3 to 29 days) and in one patient from the ganciclovir group (diagnosed 3 days after inclusion). Study treatment was stopped in 3 of 5 patients receiving placebo and in the patient from the ganciclovir group. These 4 patients were subsequently treated with open-labeled ganciclovir. Patients’ clinical courses, as assessed by temperature, white blood-cell count, platelet count, serum creatinine, radiologic score and modified clinical pulmonary infection score from randomization to day 14 as well as by SOFA score and partial oxygen pressure in arterial blood/fraction of inspired oxygen (PaO2/FiO2) ratio kinetics from randomization to day 28 were also comparable for the two study groups (see Fig. 3a and b and in the Additional file 3: Figs. S1–S6).

### Safety

The adverse event rates were comparable for the two groups: 48.7% for ganciclovir-treated patients and 48.6% placebo recipients. No leucopenia or thrombocytopenia was reported. Only one patient (from the ganciclovir group) experienced renal failure requiring renal replacement therapy. Cardiac arrest related to a torsade de pointe was identified in the ganciclovir group. Creatinine levels, white blood cells counts and platelet counts from randomization to day 14 did not differ significantly between the two groups (see in the Additional file 3: Figs. S2–S4), and the percentages of patients requiring renal replacement therapy from randomization to the end of treatment were similar (Table 3).

## Discussion

The results of this study, designed to assess the efficacy of preemptive ganciclovir for CMV reactivation in non-immunosuppressed mechanically ventilated ICU patients, show that this strategy was unable to significantly increase the number of ventilator-free days at day 60.

The incidence of CMV infection in non-immunocompromised ICU patients has been assessed in many studies and analyzed in meta-analyses [4, 30–34]. In seropositive patients monitored three times a week using quantitative plasma CMV PCR and presenting with a large variety of critical illnesses, such as sepsis, cardiac failure, burns and trauma, one-third of these patients presented with positive viremia, and the median time to the first detection of viremia was 12 days, with a range of 3 to 57 days [33]. The effects of CMV reactivation on ICU mortality and duration of mechanical ventilation are a matter of debate. In ARDS patients presenting with septic shock, an association has been found between CMV serological status and the number of ventilator-free days to day 28 [35]. Other epidemiological studies and some meta-analyses have looked more specifically at the effects of CMV infection on mortality. In particular, some recent meta-analyses have suggested that there is a strong relationship between the presence of an active CMV infection and mortality, with an odds ratio of approximately 2 [4, 30–32, 34]. In a matched cohort study [11], in which patients were matched on age, gender, SAPS II score and reason for admission, ICU mortality was higher in patients presenting with positive antigenemia (50%) compared with control patients (28%, *P* < 0.02). Increased mortality at day 60 related to CMV infection has also been reported in non-immunocompromised ICU patients (55% vs. 20%, *P* = 0.01) [30]. However, in a series of 86 patients who presented with severe sepsis, although reporting a longer duration of ICU and hospital stays and a prolonged duration of mechanical ventilation when active CMV infection was diagnosed, the authors did not report any increase in mortality related to CMV [36]. We compiled the two randomized controlled trials evaluating prophylactic ganciclovir/valganciclovir [14, 15] and the present one evaluating ganciclovir as preemptive therapy. As shown in Additional file 3: Figure S7, these meta-analyses did not show any efficacy of ganciclovir/valganciclovir in decreasing neither mortality at day 28 nor hospital mortality. In contrast, there was a beneficial effect of ganciclovir/valganciclovir in increasing the number of ventilator-free days at day 28 (Additional file 3: Fig. S7).

While prophylactic ganciclovir was associated with a statistically significant increase in ventilator-free days, Limaye et al. did not identify any significant differences between ganciclovir and placebo overall mortality, secondary bacteremia or fungemia, or ICU or hospital length of stay [15]. In this latter study [15], there was a high rate of local lung CMV reactivation in the placebo group and a significant reduction in lung viral load in the ganciclovir group, suggesting that the attenuation of CMV-mediated lung injury is a potential mechanism to explain the significant increase in ventilator-free days among patients in the ganciclovir group and a reduction in the duration of mechanical ventilation days among the group of patients who survived for at least 28 days [15]. As in the present study, it is unlikely that the increase in the number of VFDs was related to a reduced incidence of bacterial/fungal infections. The objective of increasing the number of VFDs by 8 days was probably adequate, as suggested by a recent report in which the mean difference in mechanical ventilation days was increased by 9 days between patients with and without CMV infection [4]. When analysis was restricted to CMV detection in blood, there was still a statistically significant difference in the length of mechanical ventilation, which was 7 days between patients with CMV infection and patients without infection [4]. Interestingly, CMV blood reactivation was not associated with higher mortality, which is consistent with the present study [4].

The assessment of ganciclovir safety in the present study did not permit the identification of any increase in myelotoxicity and renal insufficiency, which is in agreement with recent reports [14, 15].

There were several strengths of the study, including the placebo-controlled, double-blind, multicenter study design; the use of quantitative DNAemia as an inclusion criterion; and the inclusion of objective and clinically relevant outcomes.

## Limitations

This study has several limitations. First, it was stopped prematurely. However, based on the study results, more than 800 patients would have been necessary to show a significant difference in VFD. Second, we used biweekly DNAemia analyses with an arbitrary threshold to characterize reactivation. It is highly probable that a screening test including tracheobronchial specimens for DNA identification would have been more sensitive. Indeed, it has been shown that lung reactivation occurs earlier than reactivation in the blood (median, 14 and 24 days, respectively) [36]. Third, we used VFDs as the main outcome and not mortality or duration of mechanical ventilation. The use of VFDs as a clinical endpoint is recommended in ICU trial design guidelines, especially when the expected difference would involve the mechanical ventilation duration [24, 37, 38], which was clearly expected. Fourth, we used ganciclovir to treat CMV reactivations. When the study was designed, this was the drug of choice for treating all CMV diseases. There are now several potent, well-tolerated, and newer CMV-active antiviral drugs that could also be considered for future studies done in ICU patients [39, 40]. Fifth, it could be questioned whether preemptive therapy, as chosen in this study, is preferable to a prophylactic strategy. Prophylaxis would be theoretically more attractive because it prevents viral reactivation with its related subsequent direct or indirect damage. Prophylaxis exposes patients to the increased risk of adverse effects, whereas treatment following reactivation minimizes the population’s exposure to drug side effects and drug interactions and may decrease pharmaceutical costs.

## Conclusion

In ICU patients mechanically ventilated for ≥ 96 h with CMV reactivation in blood, preemptive ganciclovir did not increase the number of ventilator-free days at day 60. Further studies are needed that could be based on the difference in VFD60 observed in the present study to calculate the number of patients to treat. These studies would require to include more than 800 patients with CMV reactivation, which is a very striving target to reach.

## Supplementary Information

**Additional file 1.** Protocol.

**Additional file 2.** Statistical analysis plan.

**Additional file 3: Table S1.** Microorganisms Responsible for Bacteremia/Fungemia Post-Randomization According to Study Group. **Table S2.** Microorganisms other than viruses Responsible for Ventilator-Associated Pneumonia Post-Randomization According to Study Group. **Figure S1.** Temperature Kinetics from Randomization to Day 14 According to Study Group. **Figure S2.** White Blood-Cell–Count Kinetics from Randomization to Day 14 according to Study Group. **Figure S3.** Platelet-Count Kinetics from Randomization to Day 14 According to Study Group. **Figure S4.** Creatinine-Level Kinetics from Randomization to Day 14 According to Study Group. **Figure S5.** Kinetics of Radiologic Score 1 from Randomization to Day 14 According to Study Group. **Figure S6.** Evolution of Modified Clinical Pulmonary Infection Score (mCPIS) 2 from Randomization to Day 14 According to Study Group. **Figure S7.** Metanalyses from randomized clinical trials evaluating the effects of prophylactic or preemptive ganciclovir/valganciclovir on mortality at day 28, hospital mortality and ventilator-free days at day 28 (VFD28).

## Data Availability

The datasets used and/or analyzed during the current study are available from the corresponding author on reasonable request.

## Abbreviations

CMV: Cytomegalovirus
ICU: Intensive care unit
HSV: Herpesvirus simplex
PCR: Polymerase chain reaction
SAPS II: Simplified Acute Physiology Score II
SOFA: Sequential Organ Failure Assessment score
VFD60: Ventilator-free days at day 60
ARDS: Acute respiratory distress syndrome
DSMB: Data Safety Monitoring Board
SHR: Subdistribution hazard ratio
95% CI: 95% Confidence interval
IQR: Interquartile range
SD: Standard deviation
NYHA: New York Heart Association
COPD: Chronic obstructive pulmonary disease
MV: Mechanical ventilation
ECMO: Extracorporeal membrane oxygenation
PaO_2_/FiO_2_: Partial oxygen pressure in arterial blood/fraction of inspired oxygen ratio
PEEP: Positive end-expiratory pressure
CPAP: Continuous positive airway pressure
