# Supplementary material for: Preemptive ganciclovir for mechanically ventilated patients with cytomegalovirus reactivation
Source: Ann Intensive Care. 2021 Feb 11;11:33. doi: 10.1186/s13613-020-00793-2 (PMC7876264; doi:10.1186/s13613-020-00793-2)
Supplement: Supplementary file 2 — Additional file 2. Statistical analysis plan. [file 13613_2020_793_MOESM2_ESM.docx]

| 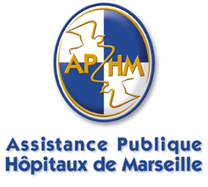 |
| --- |

**Preemptive Ganciclovir for Mechanically Ventilated Patients with Cytomegalovirus Reactivation**

**PTH CMV study**

**Multicenter, randomized and double-blinded study**

**STATISTICAL ANALYSIS PLAN**

V1.1 May 2020 (translation 20/10/2018 French version)

| **Sponsor** | Assistance publique – Hôpitaux de Marseille |
| --- | --- |
| **Principal investigator** | Prof. Laurent Papazian  Réanimation – Détresses Respiratoires et Infections Sévères  URMITE CNRS-UMR 7278 – Aix-Marseille Université |
| **Statistical supervisor** | Dr Karine Baumstarck and Anderson Loundou  Service d’Epidémiologie et Economie de la Santé  DRC AP-HM (Pr Pascal Auquier) |
| **Study protocol** | PTH Preemptive Treatment for Herpesviridae  V4.0 28/5/2015 |
| **ClinicalTrials.gov Identifier** | NCT02152358 |

**Table of Contents**

[I. Administrative aspects 3](#_Toc42240481)

[II. Hypothesis and objectives 4](#_Toc42240482)

[1. Hypothesis 4](#_Toc42240483)

[2. Study objectives 4](#_Toc42240484)

[2.1. Main objective 4](#_Toc42240485)

[2.2. Secondary objectives 5](#_Toc42240486)

[III. Methods 6](#_Toc42240487)

[1. Study design 6](#_Toc42240488)

[2. Randomisation 6](#_Toc42240489)

[3. Patients 6](#_Toc42240490)

[3.1. Inclusion criteria 6](#_Toc42240491)

[3.2. Non-inclusion criteria 6](#_Toc42240492)

[4. Sample size 7](#_Toc42240493)

[5. Interim analyses and final analysis 7](#_Toc42240494)

[6. Protocol summary 7](#_Toc42240495)

[IV. Statistical analysis 8](#_Toc42240496)

[1. Quality control of the database 8](#_Toc42240497)

[2. General issues 8](#_Toc42240498)

[3. Flow chart 8](#_Toc42240499)

[4. Study population 9](#_Toc42240500)

[5. Characteristics of the patients 9](#_Toc42240501)

[V. Statistical analysis 10](#_Toc42240502)

[1. Primary endpoint 10](#_Toc42240503)

[2. Primary endpoint : secondary analyses 11](#_Toc42240504)

[2.1. VFD D60 comparison between groups (Student ou Mann-Whitney) 11](#_Toc42240505)

[2.2. Adjustment for variables at inclusion 11](#_Toc42240506)

[2.3. Méthode des paires (Finkelstein) 12](#_Toc42240507)

[3. Secondary endpoint 12](#_Toc42240508)

[4. Safety 13](#_Toc42240509)

[5. Biological, hemodynamic and respiratory data 13](#_Toc42240510)

[6. Post-hoc analyses 14](#_Toc42240511)

# Administrative aspects

**Study protocol**

| **Main steps of PTH-CMV study** | **Date** |
| --- | --- |
| Year of funding (PHRC) | 2011 |
| Ethic committee | 14/09/2012 |
| No EUDRACT | 2012-003312-30 |
| Start of inclusions | 05/02/2014 |
| End of follow-uo | 23/03/2019 |

**SAP versions**^[[1]](#footnote-1)^

| **Numéro** | **Date** | **Justification** |
| --- | --- | --- |
| V0 | 2015, August | Short version SAP (protocol study) |
| V1.0 | 2018, October | Harmonization of PTH-CMV SAP with PTH-HSV SAP |
| V1.1 | 2020, May | Translation from French to English versions |

**Contributors**

| **Nom** | **Fonction** | **Rôle** | **Signature** |
| --- | --- | --- | --- |
| Laurent Papazian | Principal investigator | SAP co-writing and validation |  |
| Karine Baumstarck | Statistical supervisor | SAP co-writing | 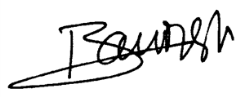 |
| Anderson Loundou | Statistician | SAP co-writing |  |

# Hypothesis and objectives

## Hypothesis

The hypothesis is that the administration of an antiviral (ganciclovir for CMV, aciclovir for HSV) as soon as a PCR is positive (blood for CMV, oropharyngeal for HSV) makes it possible to increase the number of ventilator-free days and alive (VFD) at D60 (number of days patients are alive and weaned from mechanical ventilation) (D1 = day of initiation of ganciclovir, aciclovir or placebo) of patients treated with ganciclovir compared to placebo on the one hand (CMV arm), patients treated with aciclovir compared to placebo on the other hand (HSV arm).

**The two hypotheses are addressed separately. This document corresponds to the statistical analysis plan concerning the CMV.**

## Study objectives

In mechanically ventilated patients for at least 96 hours, to show that treatment with ganciclovir in those with CMV replication reduces the duration of mechanical ventilation and improves the prognosis.

### 2.1. Main objective

To show that a 14 day treatment of ganciclovir when CMV blood PCR is positive increases the number of days that the patients are alive and weaned from mechanical ventilation on D60 after inclusion (ventilation-free days at day-60, VFD D60). The number of days without mechanical ventilation is counted as soon as there is an absence of any invasive mechanical ventilation for at least 48 hours.

The following elements are taken from the protocol:

The weaning period must be greater than 48 h without invasive mechanical ventilation to be taken into account. Patients who died before D60 have, by definition, no living day without mechanical ventilation even if they have been weaned for a few days [David A. Schoenfeld, PhD; Gordon R. Bernard, MD; for the ARDS Network. Statistical evaluation of ventilator-free days as an efficacy measure in clinical trials of treatments for acute respiratory distress syndrome. Crit Care Med 2002; 30:1772–1777].

Some examples:

- Patient weaned on D10 post-inclusion: VFD D60 = 60 - 10 = 50

- Patient weaned on D70 post-inclusion: VFD D60 = 0

- Patient weaned from D12 to D20 but died at D40: VFD D60 = 0

- Patient weaned on D45 but died on D80: VFD D60 = 60 - 45 = 15.

In the event that a patient has several periods of at least 48 hours without ventilation, VFD J60 is calculated from the last withdrawal from mechanical ventilation.

For example :

- Patient weaning n ° 1 on D5, back to invasive mechaniocal ventilation on D10 and new weaning on D28: VFD D60 will be 60 - 28 = 32.

The 60-day delay after inclusion (frequently used in ICU studies) takes into account the fact that the impact on the prognosis could go through an increase in the number and severity of bacterial infections consecutive to virus-induced immunosuppression. Beyond J60, other confounding factors can make it difficult to interpret the results.

### 2.2. Secondary objectives

To show the benefit of a 14-day treatment of ganciclovir for a CMV blood PCR on:

- Mortality on D60, on D28

- ICU mortality

- Hospital mortality

- Duration of invasive mechanical ventilation

- Duration of ICU stay

- Duration of hospitalization

- Incidence of active CMV infections - Reactivation rate

- Incidence of herpetic bronchopneumonia

- Evolution of organ failures on D3, D5, D7, D14, D21 and D28 evaluated by the SOFA score

- Incidence of bacterial infections (ventilator-associated pneumonia, bacteremia)

- Incidence of ARDS

- Incidence of septic shock

- Disappearance of CMV replication in the blood

- Tolerance of ganciclovir: incidence of myelotoxicity, renal failure

# Methods

## Study design

It is a prospective study divided into two arms, randomized, comparative, double-blinded on two times 2 parallel groups.

This SAP corresponds to the CMV arm: an experimental group using ganciclovir and a control group with standard management and a placebo in the presence of CMV-positive PCR at the blood level.

## Randomisation

Randomization is performed independently for each arm of the study (CMV and HSV). The inclusion ratio is 1: 1. Three randomization criteria are used: the center, the duration of ventilation prior to inclusion (2 levels: [4-14 days], [15-30 days]) and the presence of organ failures (2 levels: <2 or ≥ 2 organ failures according to SOFA). Allocation to a therapeutic group will be done using a minimization technique. Randomization is electronic (hotline 7 days a week and H24).

## Patients

### 3.1. Inclusion criteria

Patients who are at least 18 years of age, mechanically ventilated for at least 96 hours with a predicted mechanical ventilation duration longer than 48 hours, have CMV-positive whole blood and provided written informed consent from the patient or his/her legally authorized representative are eligible for enrollment.

### 3.2. Non-inclusion criteria

Exclusion criteria are as follows: age < 18 years; patients deprived of freedom or under legal protection; patients not covered by social security; use of acyclovir, ganciclovir or another antiviral with anti-HSV/CMV activity (e.g., cidofovir or foscarnet) at the time of randomization; patients with known hypersensitivity to ganciclovir; patients who had an active HSV or CMV infection treated during the preceding month; patients who were pregnant or lactating; patients with pancytopenia, neutropenia ≤ 500/mm3, or thrombocytopenia < 25 G/L; patients with solid-organ or bone-marrow transplants; patients on immunosuppressant therapy (including corticosteroids at ≥ 0.5 mg/kg/day of prednisone or its equivalent for >1 month); patients with human immunodeficiency virus infection; patients with moribund conditions defined as a preinclusion Simplified Acute Physiology Score (SAPS) II ≥ 75; patients regarding whom a decision had been made to withhold or withdraw life-sustaining treatment; and patients with an ICU readmission during the same hospital stay.

## Sample size

We hypothesize that a 14-day treatment with ganciclovir as soon as a “positive” CMV PCR is obtained would make it possible to obtain an 8-day increase in the number of ventilator-free days compared to placebo group. According to literature, the variability in the number of ventilator-free days of patients corresponding to the inclusion criteria for CMV has a standard deviation of 20 days.

Considering these hypotheses, in a bilateral situation and with an expected power set at 80%, the number of subjects to be included per group is 97 patients. However, it is necessary to adjust this number of subjects required by the asymptotic relative efficiency coefficient for the Mann-Whitney test (the distribution is not Gaussian) (Hollander M, Wolfe D. Nonparametric statistical methods, 2nd edition New York NY, John Wiley and Sons, Inc, 1999). The correction coefficient applied is 0.864. The number of subjects per group is therefore 112 (97 / 0.864) per group. In order to ensure that 112 observations per group will be obtained and to prevent possible secondary attrition, 120 patients will be included per treatment group. Finally, 240 patients will be included in the CMV arm (120 will receive ganciclovir, 120 will receive placebo).

## Interim analyses and final analysis

No interim analysis is planned. The final analysis will be carried out once all the inclusions will be done and once the follow-up time for the last subject included has passed. The maximum follow-up for any patient is 60 days after inclusion (randomization).

## Protocol summary

|  | **Screening**  **D4-D30** | **Inclusion** | **D3** | **D5** | **D7** | **D14** | **D21** | **D28** | **End of treatment*** | **D60** |
| --- | --- | --- | --- | --- | --- | --- | --- | --- | --- | --- |
| **Inclusion criteria** | X | X |  |  |  |  |  |  |  |  |
| **SOFA/CPIS** |  | X | X | X | X | X | X | X | X |  |
| **Platelets/WBC/creatinine** |  | X | X |  | X | X |  |  | X |  |
| **Pregnancy test** |  | X |  |  |  |  |  |  |  |  |
| **PCR** | X | 2 times a week until ICU discharge or D30 maximum | | | | | | |  |  |
| **Outcomes** |  |  |  |  |  |  |  |  | X | X |

* If stopped prior D14

# Statistical analysis

## Quality control of the database

The quality control of the database will be carried out by the CHU of Angers (Data management and evaluation unit - CHU Angers) in connection with the Data review report. Particular attention will be paid to the main evaluation criterion.

Statistical processing will only be started after checking the validity of the database (issuing requests to the clinicians involved in the study, consistency checks). There is a data anonymization procedure and algorithm that assigns each individual a number. After freezing the database, the consolidated data will be processed by the statistician.

## General issues

Data analysis will be carried out using SPSS software version 17.0 under windows, by the statistician (Anderson Loundou, Department of Epidemiology and Health Economics, AP-HM) under the responsibility of Pr Pascal Auquier and the referent physician Dr Karine Baumstarck. The significance threshold will be set at 0.05. No interim analysis is scheduled. The analysis will be performed blindly; the statistician will not know the identification of the groups and will first produce the results in this form for the coordinating investigator and the associated investigators. Once the analysis is finalized, the identification of the groups will be provided. The methodology and analysis plan are based on the criteria developed by the group the Consolidated Standards of Reporting Trials Statement (CONSORT, http: // [www.consort-statement.org/consort-statement/](http://www.consort-statement.org/consort-statement/)).

## Flow chart

A figure will summarize the number of eligible subjects, the number of subjects included, the number of randomized subjects, the number of subjects followed at the different assessment times as well as the reasons for the main cases of lost contact and non-inclusions. The number of subjects at the different stages of the study will be presented using the following model (CONSORT 2010 Flow Diagram):


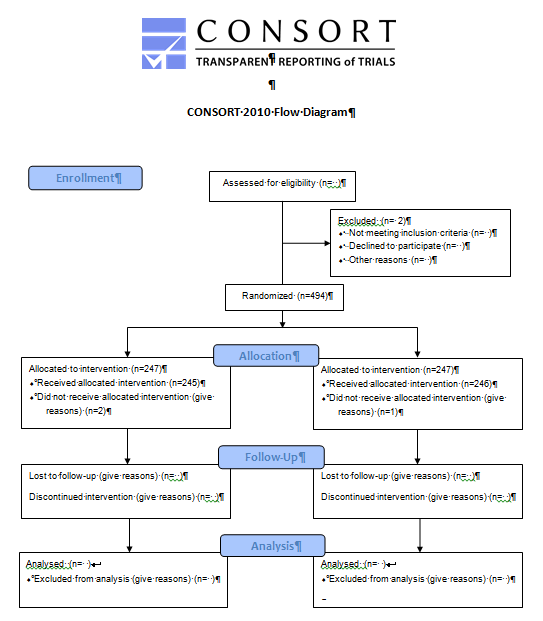


## Study population

The statistical analysis will focus on the intention-to-treat population (main analysis).

For documentary purposes, an additional analysis will be carried out in the population per protocol (secondary analysis).

## Characteristics of the patients

The number of eligible subjects, the number of subjects included and the ratio included / eligible will be provided. The number of inclusions per center will be provided (total, per group).

As a first step, a descriptive analysis covering the entire sample included will be carried out (intention-to-treat population); the variables collected at inclusion and on admission will be reported.

Qualitative variables will be presented as proportions and numbers, quantitative variables using means and standard deviation, or median and quartiles. The proportion of missing data will be specified for each variable. The normality of these parameters will be assessed using frequency histograms and Shapiro tests; simple mathematical transformations can be used to normalize non-normal data.

The results will also be produced by group: "experimental" and "control". As recommended by the CONSORT group, the comparison of the 2 groups on the variables collected at inclusion will not be provided. It will nevertheless be carried out to ensure the comparability of the groups on the main inclusion criteria.

# Statistical analysis

## Primary endpoint

The primary endpoint is the number ventilator-free days at D60: ventilator-free days and alive (VFD).

The number of VFDs will be provided for the 2 groups "experimental" versus "control": mean and standard deviation, median and interquartile range, minimum and maximum.

- **Analyses**

The main analysis will use the competitive risk method using the Fine and Gray model (Fine, J, Gray RJ. A proportional hazards model for subdistribution of a competing risk. Journal of the American Statistical Association. 1999; 94 (446): 496–509). Competitive events will be taken into account by defining weaning from mechanical ventilation as an event of interest and death as a competitive event (composite criterion "alive and free of invasive mechanical ventilation"). For the composite criterion, the results will be provided as Hazard Ratio (subdistribution hazard ratio SHR) and the 95% confidence interval (95% CI), and effect size (ES). The cumulative incidence functions will be illustrated in the form of a graph.

For each element of the composite criterion (mortality and duration of ventilation), the parameters HR and 95% CI, ES will be produced independently to document the share of each in the effect of the composite criterion. The cumulative bearing functions will also be produced.

- **Method to calculate the number of ventilator-free days**

VFD D60 will be calculated taking into account the weaning date and the notion of death or not before D60.

- The counting of days is initiated from the day of randomization: day 0.

- Successful weaning from mechanical ventilation is defined by the absence of reventilation within 48 hours of weaning.

- Patients who died before D60 have a VFD D60 = 0;

- Patients who did not die on D60 but are still invasively ventilated after D60 have a VFD D60 = 0;

- Patients not deceased on D60 and not ventilated on D60 have a VFD D60 = 60-day of weaning.

- In the event that a patient has several periods of at least 48 hours without ventilation, VFD J60 is calculated from the last withdrawal from mechanical ventilation.

Examples of calculation:

Patient weaned on D10 post-inclusion: VFD D60 = 60 - 10 = 50.

- Patient weaned on D70 post-inclusion: VFD D60 = 0.

- Patient weaned from D12 to D20 but died at D40: VFD D60 = 0.

- Patient weaned on D45 but died on D80: VFD D60 = 60 - 45 = 15.

- Patient weaning n ° 1 on D5, back to invasive mechanical ventilation on D10 and new weaning on D28: VFD D60 will be 60 - 28 = 32.

This approach will be the primary analysis for the primary endpoint.

## Primary endpoint : secondary analyses

To complement the competitive risk method (primary analysis, see above), additional analyses will be performed (secondary analyses).

### 2.1. VFD D60 comparison between groups (Student ou Mann-Whitney)

The number of VFD D60 will be compared between groups ("experimental" versus "control"):

- normal distribution (or possible normalization) : Student t test

- non normal distribution : Mann-Whitney test

### 2.2. Adjustment for variables at inclusion

Adjustment for potential confounding factors will be performed (centre, SOFA score, SAPS II, age, mechanical ventilation duration before inclusion, McCabe score, type of admission, mechanical ventilation reason, ECMO, plateau pressure, PaO2/FiO2, temperature, radiologic score radio, mCPIS). Two possible situations :

- normal distribution (or possible normalization) : ANOVA-ANCOVA
- non normal distribution : ANOVA-ANCOVA (confirmed by Mann-Whitney test)

### 2.3. Matched pairs method (Finkelstein)

VFD D60 may be analysed from other methods, taking account the death [Fish 2014, Beitler 2019]. The method remains on the postulate that a deceased patient is a more severe case that a survival patient [Finkelstein 1999] : hierarchical composite endpoint (alive and ventilation free) that treats death as a worse than prolonged ventilation, and compares each patient with every other patient in a win-lose-tie for each comparison (Mann-Whitney test).

For each comparison, a score is provided to each patient as follows :

- Pair including a deceased patient and an alive patient : score -1 and score +1, respectively
- Pair including two alive patients : score +1 for the patient who has the higher VFD D60 and score -1 for the second patient
- Pair including two alive patients with equal VFD D60 : score 0 for the two patients
- Pair including two deceased patients : score 0 for the two patients.

For each patient, the sum of the scores is calculated. The score sums will be compared between the 2 groups.

Fish E, Novack V, Banner-Goodspeed VM, Sarge T, Loring S, Talmor D. The Esophageal Pressure-Guided Ventilation 2 (EPVent2) trial protocol: a multicentre, randomised clinical trial of mechanical ventilation guided by transpulmonary pressure. BMJ Open. 2014 Oct 6;4(9):e006356.

Beitler JR, Sarge T, Banner-Goodspeed VM, Gong MN, Cook D, Novack V, Loring SH, Talmor D; EPVent-2 Study Group. Effect of Titrating Positive End-Expiratory Pressure (PEEP) With an Esophageal Pressure-Guided Strategy vs an Empirical High PEEP-Fio2 Strategy on Death and Days Free From Mechanical Ventilation Among Patients With Acute Respiratory Distress Syndrome: A Randomized Clinical Trial. JAMA. 2019 Feb 18. doi: 10.1001/jama.2019.0555.

Finkelstein DM, Schoenfeld DA. Combining mortality and longitudinal measures in clinical trials. Stat Med. 1999 Jun 15;18(11):1341-54.

## Secondary endpoint

The secondary endpoints will be compared between groups.

Chi2 test (or Fisher exact test) for :

- Death at D60
- ICU mortality
- Hospital mortality
- Delay from ICU admission and CMV reactivation
- CMV viral load
- Co-reactivation HSV at inclusion
- HSV bronchopneumonitis (aciclovir treatment, duration)
- Cytomegalovirus infection (site, treatment, duration)
- Ventilator-associated pneumonia, bacteremia or fungemia
- ARDS post-randomization
- Septic shock post-randomization
- Renal replacement therapy

Student t test (or Mann-Whitney test) for:

- Mechanical ventilation duration
- ICU stay length
- Hospitalization length
- Number of days with study drug
- Number of days with organ failure (SOFA score) from inclusion and D28

Kaplan Meier method and logrank test:

- time to death (from randomization)
- time to weaning-of mechanical ventilation (from randomization)
- time to ARDS (from randomization)
- time to septic shock (from randomization)

## Safety

The adverse event rates will be provided by « organ », will be described as numbers (percentage) for each treatment group. Comparisons between groups will performed using Chi2 test (or Fisher test).

## Biological, hemodynamic and respiratory data

Analysis of temperature, mCPIS, radiologic score, platelets, white blood cells, and creatinine levels will be resumed at randomisation, D3, D7 and D14 (mean, median, standard deviation, IQR, minimal and maximal values) according to treatment group.

Analysis of PaO2/FiO2 and SOFA score will be resumed at admission, randomisation, D3, D5, D7, D14, D21 and D28 (mean, median, standard deviation, IQR, minimal and maximal values) according to treatment group.

## Post-hoc analyses (optional)

The center effect on the primary endpoint will be assessed using a mixed effects modeling (with center as a random effect) using generalized linear mixed model (GLIMMIX procedure; center as a random effect; logit link function; binomial distribution function); the result was presented as the odd ratio and its 95% CI.

1. JAMA [Gamble C, Krishan A, Stocken D, et al. Guidelines for the Content of Statistical Analysis Plans in Clinical Trials. JAMA. 2017;318(23):2337–2343. doi:10.1001/jama.2017.18556] [↑](#footnote-ref-1)
