# Supplementary material for: Preemptive ganciclovir for mechanically ventilated patients with cytomegalovirus reactivation
Source: Ann Intensive Care. 2021 Feb 11;11:33. doi: 10.1186/s13613-020-00793-2 (PMC7876264; doi:10.1186/s13613-020-00793-2)
Supplement: Supplementary file 3 — Additional file 3: Table S1. Microorganisms Responsible for Bacteremia/Fungemia Post-Randomization According to Study Group. Table S2. Microorganisms other than viruses Responsible for Ventilator-Associated Pneumonia Post-Randomization According to Study Group. Figure S1. Temperature Kinetics from Randomization to Day 14 According to Study Group. Figure S2. White Blood-Cell–Count Kinetics from Randomization to Day 14 according to Study Group. Figure S3. Platelet-Count Kinetics from Randomization to Day 14 According to Study Group. Figure S4. Creatinine-Level Kinetics from Randomization to Day 14 According to Study Group. Figure S5. Kinetics of Radiologic Score 1 from Randomization to Day 14 According to Study Group. Figure S6. Evolution of Modified Clinical Pulmonary Infection Score (mCPIS) 2 from Randomization to Day 14 According to Study Group. Figure S7. Metanalyses from randomized clinical trials evaluating the effects of prophylactic or preemptive ganciclovir/valganciclovir on mortality at day 28, hospital mortality and ventilator-free days at day 28 (VFD28). [file 13613_2020_793_MOESM3_ESM.docx]

**Preemptive Ganciclovir for Mechanically Ventilated Patients with Cytomegalovirus Reactivation**

A Randomized Clinical Trial

Laurent Papazian, M.D., Ph.D., Samir Jaber, M.D., Ph.D., Sami Hraiech, M.D., Ph.D., Karine Baumstarck, M.D., Ph.D., Sophie Cayot-Constantin, M.D., Nadia Aïssaoui-Balanant, M.D., Ph.D., Boris Jung, M.D., Ph.D., Marc Leone M.D., Ph.D., Elisabeth Coupez, M.D., Carole Schwebel, M.D., Ph.D., Jérémy Bourenne, M.D., Jérôme Allardet-Servent, M.D., Thierry Boulain M.D., Ph.D., Qin Lu, M.D., Ph.D., Christine Zandotti, M.D., Ph.D., Anderson Loundou, M.Sc., Christine Penot-Ragon, Ph.D., Jean Chastre, M.D., Jean-Marie Forel, M.D., Ph.D., Charles-Edouard Luyt, M.D., Ph.D. on behalf of the Preemptive *Herpesviridae* Treatment Study Group, REVA Network

**Table S1. Microorganisms Responsible for Bacteremia/Fungemia Post-Randomization According to Study Group**.

| **Parameter** | **Placebo group**  **(N=37)** | **Ganciclovir group**  **(N=39)** |
| --- | --- | --- |
| Bacteremia |  |  |
| Enterobacteriaceae | 3 (8) | 8 (21) |
| *Pseudomonas aeruginosa* | 1 (3) | 3 (8) |
| *Stenotrophomonas maltophilia* | 1 (3) | 1 (3) |
| *Acinetobacter* spp. | 0 (0) | 1 (3) |
| Coagulase-negative staphylococci | 1 (3) | 0 (0) |
| *Staphylococcus aureus* | 1 (3) | 0 (0) |
| Other cocci | 3 (8) | 1 (3) |
| *Bacteroides* spp. | 0 (0) | 1 (3) |
| Fungemia |  |  |
| Other than *Candida* spp. | 0 (0) | 1 (3) |

Results are expressed as No. (%).

**Table S2. Microorganisms other than viruses Responsible for Ventilator-Associated Pneumonia Post-Randomization According to Study Group**.

| **Parameter** | **Placebo group**  **(N=37)** | **Ganciclovir group**  **(N=39)** |
| --- | --- | --- |
| Bacteria |  |  |
| Enterobacteriaceae | 5 (14) | 9 (23) |
| *Pseudomonas aeruginosa* | 9 (24) | 6 (15) |
| *Stenotrophomonas maltophilia* | 3 (8) | 1 (3) |
| *Acinetobacter* spp. | 0 (0) | 1 (3) |
| *Streptococcus pneumoniae* | 1 (3) | 0 (0) |
| Coagulase-negative staphylococci | 1 (3) | 0 (0) |
| *Staphylococcus aureus* | 1 (3) | 3 (8) |
| Other cocci | 4 (11) | 5 (13) |
| Fungi |  |  |
| *Candida* spp. | 2 (5) | 0 (0) |
| Other | 1 (3) | 0 (0) |

Results are expressed as No. (%).

**Figure S1. Temperature Kinetics from Randomization to Day 14 According to Study Group.**


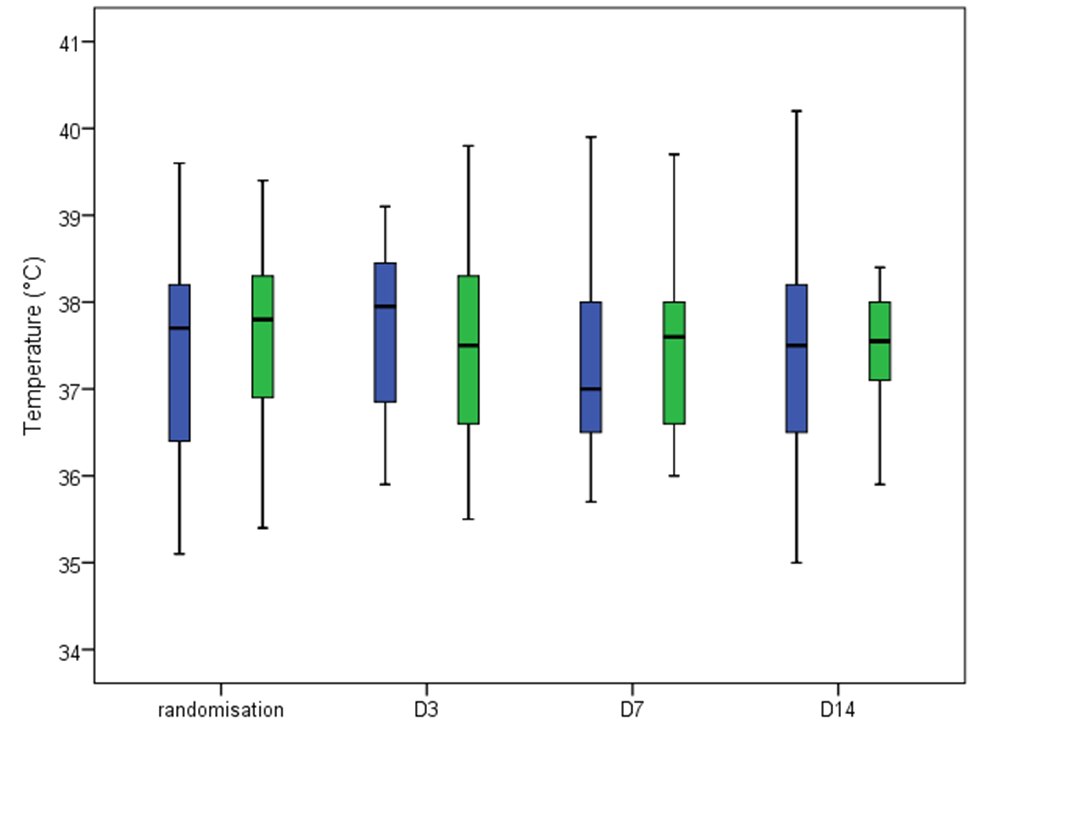


Placebo

Ganciclovir

**Figure S2. White Blood-Cell–Count Kinetics from Randomization to Day 14 according to Study Group.**


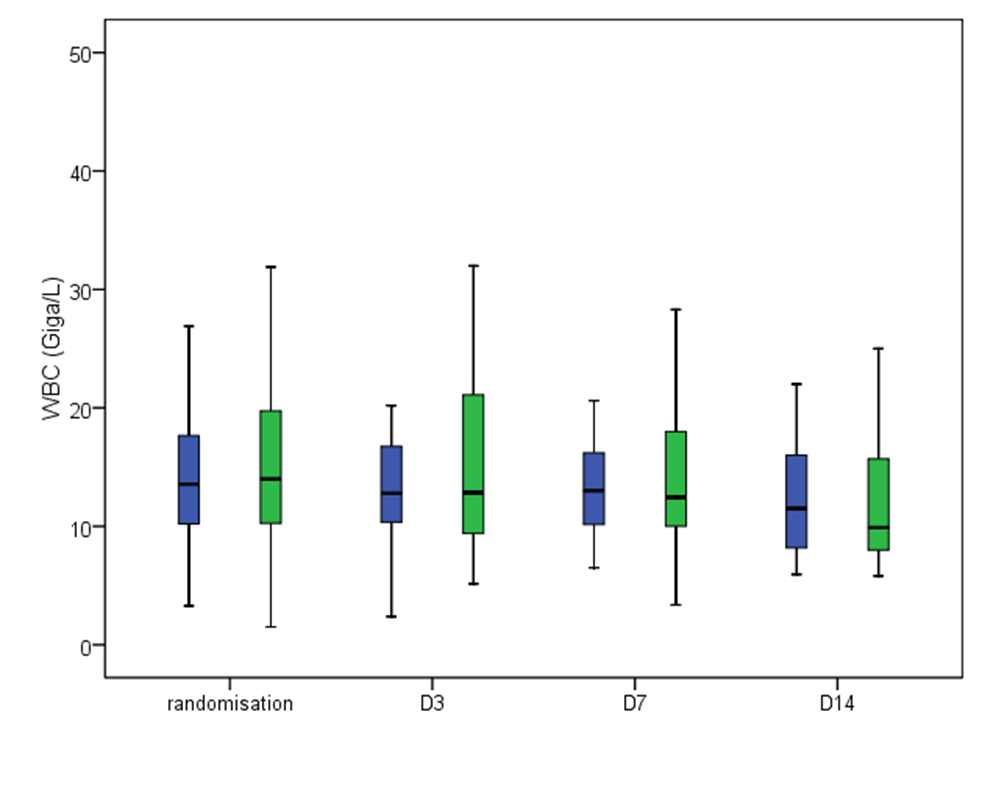


Placebo

Ganciclovir

**Figure S3. Platelet-Count Kinetics from Randomization to Day 14 According to Study Group.**

**
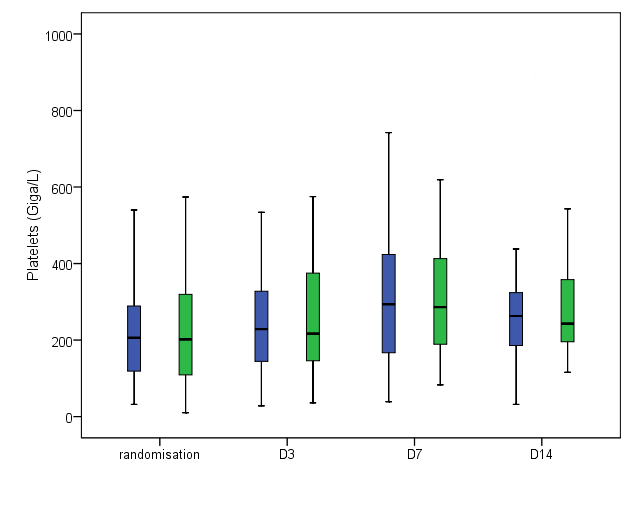
**

Placebo

Ganciclovir

**Figure S4. Creatinine-Level Kinetics from Randomization to Day 14 According to Study Group**

**
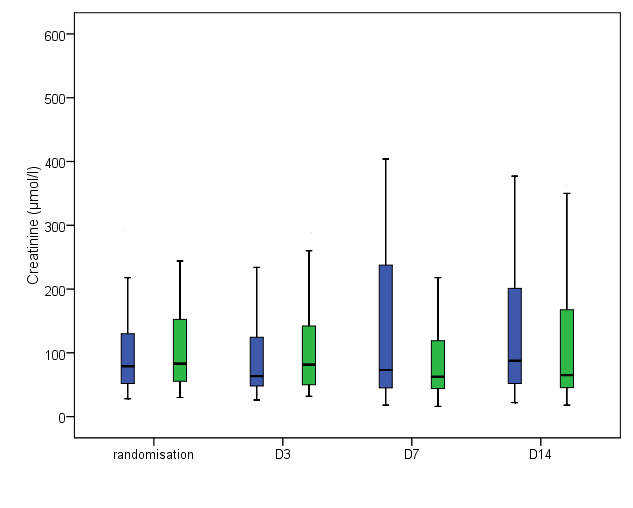
**

Placebo

Ganciclovir

**Figure S5. Kinetics of Radiologic Score ^1^ from Randomization to Day 14 According to Study Group.**


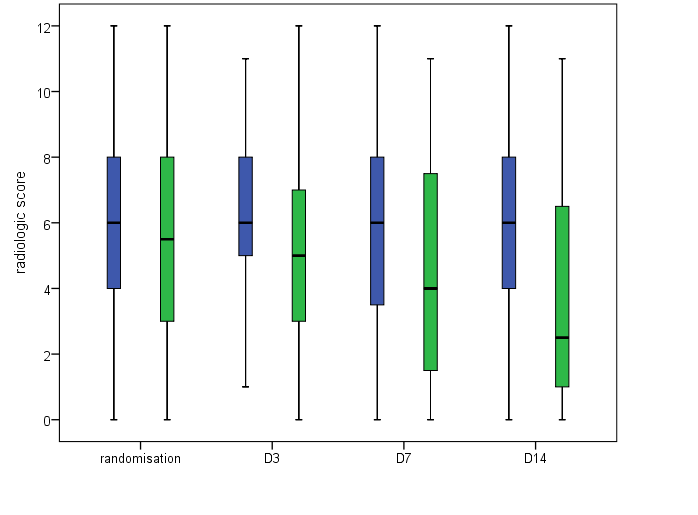


Placebo

Ganciclovir

**Figure S6. Evolution of Modified Clinical Pulmonary Infection Score (mCPIS) ^2^ from Randomization to Day 14 According to Study Group.**


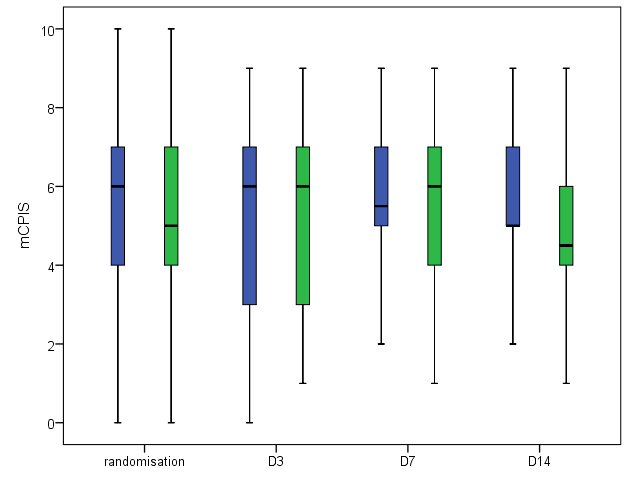


Placebo

Ganciclovir

**Figure S7. Metanalyses from randomized clinical trials evaluating the effects of prophylactic or preemptive ganciclovir/valganciclovir on mortality at day 28, hospital mortality and ventilator-free days at day 28 (VFD28)**

# References

1. Weinberg PF, Matthay MA, Webster RO, Roskos KV, Goldstein IM, Murray JF. Biologically active products of complement and acute lung injury in patients with the sepsis syndrome. *Am Rev Respir Dis.* 1984;130(5):791-796.

2. Luna CM, Blanzaco D, Niederman MS, et al. Resolution of ventilator-associated pneumonia: prospective evaluation of the clinical pulmonary infection score as an early clinical predictor of outcome. *Crit Care Med.* 2003;31(3):676-682.
